# Enterovirus A71 infection-induced dry eye-like symptoms by damaging the lacrimal glands

**DOI:** 10.3389/fcimb.2024.1340075

**Published:** 2024-04-02

**Authors:** Nan Zhou, Taige Chen, Qiao You, Deyan Chen, Lifei Liu, Kai Hu

**Affiliations:** ^1^ Department of Ophthalmology, Affiliated Drum Tower Hospital, Medical School of Nanjing University, Nanjing, China; ^2^ Department of Rheumatology and Immunology, Affiliated Drum Tower Hospital, Medical School of Nanjing University, Nanjing, China; ^3^ Center for Public Health Research, Medical School of Nanjing University, Nanjing, China; ^4^ Department of Infectious Disease, Children’s Hospital of Nanjing Medical University, Nanjing, China

**Keywords:** EV-A71, hand, foot and mouth disease (HFMD), ocular surface, dry eye, lacrimal gland

## Abstract

**Purpose:**

To determine the effects of EV-A71 (Enterovirus A71) infection on ocular surface and its mechanism.

**Methods:**

AG6 mice aged two to three weeks were randomly divided into control and EV-A71 infected groups. Slit-lamp observation, fluorescein staining, and phenol red thread test were used to assess symptoms of ocular surface at 4 dpi (days post infection). The pathological changes of cornea and lacrimal gland were observed by H&E staining, PAS staining, TUNEL assay, IHC staining and qRT-PCR. Corneas and lacrimal glands from mice were obtained and processed for RNA sequencing analysis. Newly diagnosed HFMD patients caused by EV-A71 were recruited and ensured they met the inclusion criteria. Ocular surface parameters (TMH and NIKBUT) were measured using the OCULUS Keratograph 5M. Tear samples were taken to examine Cxcl1 and IL-6 levels through the ELISA method.

**Results:**

Mice studies revealed that EV-A71 infection caused tear film instability, decreased tear secretions, decreased in lacrimal gland size, and distinct goblet cell loss. It also resulted in increased large vacuoles within acinar cells and structural damage in lacrimal gland. Apart from minor damage to the epidermis, there was no obvious inflammatory changes or apoptosis in the cornea. However, there were significant inflammatory injury and apoptosis in the lacrimal gland. RNA-seq analysis showed IL-17 and NF-κB signaling pathways were activated in the lacrimal glands of mice infected with EV-A71. In HFMD patients, the THM was in a low range and NITBUT was significantly shorter than the control group by Oculus Keratograph 5M. ELISA assay showed a higher tear Cxcl1 and IL-6 level in them.

**Conclusion:**

EV-A71 infection affected lacrimal gland structure and function and induced dry eye-like symptoms.

## 1 Introduction

Hand, foot and mouth disease (HFMD) is a highly contagious disease commonly seen in young children, characterized by typical manifestations such as oral herpes and rashes on the hands and feet (Zhu et al., 2023). The disease was generally mild and lasted less than one week in most cases (Lim et al., 2020). Enterovirus A71 (EV-A71) and Coxsackievirus A16 (CV-A16) are the predominant viruses causing HFMD worldwide (Aswathyraj et al., 2016). HFMD currently presents a major threat to infants and young children, becoming a significant concern for public health throughout the Asia–Pacific region and beyond (Puenpa et al., 2019; Yan et al., 2022). Enterovirus 71 (EV-A71), first isolated in 1969, is a positive single-stranded RNA virus from the enterovirus genus of the picornaviridae family (Brown and Pallansch, 1995; Solomon et al., 2010). According to the epidemiological study, EV-A71 was the predominant virus in the > 2 years old groups in Mainland China (Liu et al., 2015). Therefore, we chose EV-A71 for research in subsequent experiments.

Dry eye is a multifactorial disease of the ocular surface characterized by a loss of homeostasis of the tear film (Clayton, 2018) or a decreased production in tears, which cause damage to the ocular surface. The lacrimal gland is the main exocrine gland of the eye and secrete lacrimal fluid as a major component of the tear film (Hayashi et al., 2022), which has a significant role in maintaining the stability of the homeostatic environment for a healthy ocular surface (Jin et al., 2020). In other words, when a loss of function occurs in the lacrimal gland, a significant reduction in tear production and dry eye may occur (Yao and Zhang, 2017). Many systemic diseases can contribute to the damage of lacrimal gland, including autoimmune diseases (Zhou et al., 2020), sleep loss (Huang et al., 2022), radiation therapy (Zhang et al., 2014) and aging (Kojima et al., 2012). Actually, virus infectious diseases, such as acquired immunodeficiency syndrome (AIDS) (Nizamuddin et al., 2018), herpes stromal keratitis (Rao et al., 2019), chronic hepatitis C (Koike et al., 1997) and Coronavirus disease 2019 (COVID-19) (Kase and Ishida, 2022; Shen et al., 2023), are also a major factor causing lacrimal gland injury and dry eye syndrome. However, the possible pathogens and pathogenic mechanisms for virus-related dry eye are largely unknown.

Only a few case reports have described eye disorders associated with HFMD, for instance, postinfectious optic neuritis (Barrett et al., 2021) and acute unilateral maculopathy (Reich et al., 2019). Therefore, the impacts of HFMD on the eye need to be more well-established and often clinically neglected. There are two main reasons why we chose to study the relationship between ocular surface and HFMD. One reason is that even relatively mild HFMD patients can elevate serum levels of inflammatory cytokines due to systemic inflammation, which may be a factor for dry eye (Zeng et al., 2013; Wang et al., 2023). Another reason is that studies have confirmed the susceptibility of lacrimal glands to some viral infections (Ohguchi et al., 2006; Montgomery et al., 2019; Nakamura et al., 2020). Considering the diversity of HFMD virus species, our study focused on exploring EV-A71. It is worth mentioning that Enterovirus D70 (EV-D70) is recognized as the main causative agent of acute hemorrhagic conjunctivitis, a highly contagious viral infection of the eye (Sattar et al., 1988). However, whether these two enteroviruses (D70 and A71) have the same underlying mechanism of action in the eye is unknown. Follow-up studies are continuing to investigate whether other types of viruses, such as coxsackie virus, cause dry eye surface symptoms.

In the current study, we explored the effects of EV-A71 infection on corneas and lacrimal glands in experimental mice model and carried out the non-invasive tear film tests in HFMD patients. The results showed that EV-A71 induced lacrimal gland inflammation and decreased tear secretion which resulted in dry eye-like symptoms. Through the approach of blending clinical observation with mice model, we illustrated the inflammatory processes involved, offering a comprehensive perspective on HFMD’s systemic impacts and guiding future clinical practice toward early detection and management of ocular symptoms.

## 2 Materials and methods

### 2.1 Patients

In this prospective study, we examined 40 eyes of 20 patients with HFMD and 36 eyes of 18 healthy children at the Nanjing Drum Tower Hospital from July 2023 to September 2023. HFMD pediatric patients were diagnosed with HFMD by an experienced doctor in the Department of Pediatric Infectious Diseases. Throat swabs were collected from HFMD cases to ensure the virus strain. This study was approved by the Ethics Committee of Children’s Hospital of Nanjing Medical University (Application No.202311015-1) and conformed to the tenets of the Helsinki Declaration. Informed consent was obtained from all patients’ parents or legal guardians.

Inclusion criteria: (1) both the diagnosis and clinical classification complied with the Diagnosis and Treatment Guidelines for Hand-Foot-Mouth Disease (2018 Edition) developed by the Ministry of Health of the People’s Republic of China; (2) the child must be able to follow the ophthalmologist’s instructions; (3) patients were positive for EV-A71 by throat swabs. Exclusion criteria: (1) systemic disease or usage of drugs that influenced the corneal epithelium; (2) a previous history of ocular surgery; (3) the presence of other ophthalmic diseases.

### 2.2 Clinical examination

Oculus Keratograph 5M (Wetzlar, Germany) can provide a simple non-invasive method of examination for dry eye. Tear film metrics were assessed with the K5M to evaluate tear meniscus height (TMH) and non-invasive Keratograph tear breakup time (NIKBUT, including NIKBUT-first and NIKBUT-average) from both eyes of each subject. The examination was performed in a dusk room by a masked ophthalmologist and the subject was confirmed not to be interfered from external factors.

### 2.3 Tear sample collection and determination of Cxcl1 and IL-6

Human tears were collected from participants by washing each eye with 1 mL of phosphate buffered saline (PBS), collecting the wash with sterile tubes, and combining the wash from the right and left eyes of individual participants (tears collected in the same tube). All tear samples were stored at -80°C until detection. The protein expressions of Cxcl1 and IL-6 were detected by Elisa using Human Cxcl1 ELISA Kit and Human IL-6 ELISA Kit (YOBIBIO, China).

### 2.4 Cells and virus

Vero cells (african green monkey kidney epithelial cells) were purchased from the American Type Culture Collection. Vero cells were grown in high-glucose DMEM (HyClone, Logan, UT, USA) plus 10% or 2% FBS (Gibco, Carlsbad, CA, USA). Cells were incubated at 37°C with a 5% CO_2_ humidified atmosphere.

The BrCr strain of EV-A71 used in this study was obtained from Dr. Bin Wu (Wang et al., 2019). Vero cells were used for EV-A71 multiplication. The virus titers were calculated as the 50% tissue culture infectious dose (TCID50) using the Reed–Munch method as previously described.

### 2.5 Animal study

The strain of mice used in this study was AG6 (IFN-α/β and IFN-γ receptor deficient). AG6 mice were kindly provided by Qibin Leng (Institute Pasteur of Shanghai, Chinese Academy of Sciences). All mice were bred under specific pathogen-free conditions in the Animal Resource Center at the Nanjing university. Both female and male mice were used in the experiment. Two-week-old AG6 mice were infected with EV-A71 (10^7^ PFU) via the intraperitoneal (i.p.) route. Eyes of the mice were observed by fluorescence sodium dyeing (Jingming, Tianjin, China), tear production was measured by phenol red-impregnated cotton threads (Jingming, Tianjin, China) and then tissues were obtained for the experiments at 4 days post-infection (dpi). All experimental procedures in animals were approved by Animal Care and Use Committee of Nanjing University and were carried out in accordance with the Association for Research in Vision and Ophthalmology (ARVO) Statement for the Use of Animals in Ophthalmic and Vision Research.

### 2.6 Measurement of corneal fluorescein staining and tear production

Corneal fluorescein staining was conducted in right eye of the infected mice at different time points. 2 μl of 0.25% fluorescein sodium was dropped topically in the conjunctival sac. After closing the mouse eyelids a few times, the eyes were rinsed with 0.9% saline and examined by the slit-lamp biomicroscope under cobalt blue light. To quantify the extent of damage to the corneal epithelium, the corneal fluorescein score (CFS) method was performed. Briefly, the cornea was divided into 4 quadrants, and the score of each quadrant was evaluated as follows: 0 points (no staining), 1 point (spot-shaped staining), 2 points (spot-shaped staining), and 3 points (flake staining). The score of each cornea was the sum of 4 quadrants’ scores.

Tear production was measured by the phenol red thread tear test using cotton thread. The threads were placed in the inferior conjunctival sac for 3 min in each eye, and the length of the tear-soaked thread was recorded by the same person each time.

### 2.7 H&E, PAS and IHC staining

Eyeballs and lacrimal gland were fixed in 4% formaldehyde and embedded in paraffin. The eyeballs tissue was sliced into 4-μM-thick sections, deparaffinized with xylene, and then hydrated in an ethanol gradient. Then, the sections were stained with hematoxylin-eosin staining kit (Servicebio, Wuhan, China). The eyeballs and eyelids collected from mice were fixed in 4% paraformaldehyde, then embedded with paraffin and sectioned into 5 µm thickness. A PAS staining kit (Servicebio, Wuhan, China) was used to stain the sections. For immunohistochemistry analysis, the sections were incubated with anti-Enterovirus A71 antibodies (Abcam, ab36367) at 4°C overnight. Subsequently, the sections were incubated with secondary antibody (Alexa Fluro 488-labeled goat anti-mouse IgG) for 30 min at room temperature. The bound antibody was performed with a DAB substrate kit (Thermo Scientific). Photos were taken of the sections using a light microscope (Leica Microsystems, Wetzlar, Germany).

### 2.8 RNA isolation and quantitative Real-Time PCR

Total RNA was extracted from cells and tissues using the Trizol reagent (Vazyme, Nanjing, China). According to our previous work, the cDNA was synthesized by reverse transcription of 1 µg of total RNA using the HiScript II Q Select RT SuperMix (Vazyme, Nanjing, China) according to the manufacturer’s instructions. qRT-PCR was performed on ABI QuantStudio 6 Flex (Invitrogen, Carlsbad, CA, United States) using the ChamQ Universal SYBR qRT-PCR Kit (Vazyme, Nanjing, China). GAPDH or β-actin was served as an internal control, and relative expressions of genes were calculated by the 2^−ΔΔCT^ method. Primer sequences are listed in **Supplementary Table 1**.

### 2.9 ELISA

HMGB1 in mice serum was quantitated by a Mouse HMGB1 ELISA Kit (S203674, D&B Biological Science and Technology, Shanghai, China) according to the manufacturers’ instructions. MRX II microplate reader (Dynex, Chantilly, VA, United States) was used to measure OD values for each well at 450 nm.

### 2.10 TUNEL assay

Tissues were removed and placed in optimum cutting temperature (OCT) glue at −80 °C overnight. Frozen sections were taken and left at room temperature for about 1 h. To detect the apoptosis of cornea and lacrimal gland, frozen sections were tested by *in situ* TUNEL assay according to the manufacturer’s instructions (Roche Diagnostics GmbH, Mannheim, Germany). Photographs were captured using the Leica Thunder system (Leica, Wetzlar, Germany).

### 2.11 Protein-protein interaction establishment and identification of hub genes

An online tool (Search Tools for the Retrieval of Interacting Genes, STRING4) was used to analyze protein interactions. The PPI pairs were screened by confidence score (>0.40), and the PPI network was visualized by the Cytoscape V3.9.0 software. MCC was calculated through CytoHubba to evaluate the importance of each node, and the top 10 nodes were selected. The hub genes were their common nodes.

### 2.12 UID RNA-seq experiment

#### 2.12.1 RNA extraction, library preparation and sequencing

Total RNAs were extracted from corneas and lacrimal glands using TRIzol Reagent (Invitrogen, USA) following the manufacturer’s procedure. DNA digestion was carried out after RNA extraction by DNaseI. RNA quality was determined by examining A260/A280 with Nanodrop™ OneCspectrophotometer (Thermo Fisher Scientific). RNA Integrity was confirmed by 1.5% agarose gel electrophoresis. Qualified RNAs were finally quantified by Qubit3.0 with QubitTM RNA Broad Range Assay kit (Life Technologies). 2 μg total RNAs were used for stranded RNA sequencing library preparation using KC-DigitalTM Stranded mRNA Library Prep Kit for Illumina® (Catalog NO. DR08502, Wuhan Seqhealth Co., Ltd. China) following the manufacturer’s instruction.

#### 2.12.2 RNA-seq data analysis

Raw sequencing data was first filtered by Trimmomatic (version 0.36), low-quality reads were discarded and the reads contaminated with adaptor sequences were trimmed. Clean Reads were further treated with in-house scripts to eliminate duplication bias introduced in library preparation and sequencing. Reads in the same cluster were compared to each other by pairwise alignment, and then reads with sequence identity over 95% were extracted to a new sub-cluster. After all sub-clusters were generated, multiple sequence alignment was performed to get one consensus sequence for each sub-clusters. After these steps, any errors and biases introduced by PCR amplification or sequencing were eliminated. The de-duplicated consensus sequences were used for standard RNA-seq analysis. Genes differentially expressed between groups were identified using the edgeR package (version 3.12.1). An FDR q-value cutoff of 0.05 and Fold-change cutoff of 2 were used to judge the statistical significance of gene expression differences. Gene ontology (GO) analysis and Kyoto encyclopedia of genes and genomes (KEGG) enrichment analysis for differentially expressed genes were both implemented by KOBAS software (version: 2.1.1) with a FDR q-value cutoff of 0.05 to judge statistically significant enrichment. The raw data were uploaded to SRA database and the BioProject ID is PRJNA1071792.

### 2.13 Statistical analysis

Statistical analyses were performed using GraphPad Prism 8.0 (GraphPad Software, San Diego, CA, USA). Each experiment was repeated at least 3 times. The results were shown as mean ± standard error of the mean (SEM) and were compared using Student t-tests. *P < 0.05 represents the statistical significance.

## 3 Results

### 3.1 Symptoms of dry eye in EV-A71 infected mice model

**Figure 1:**
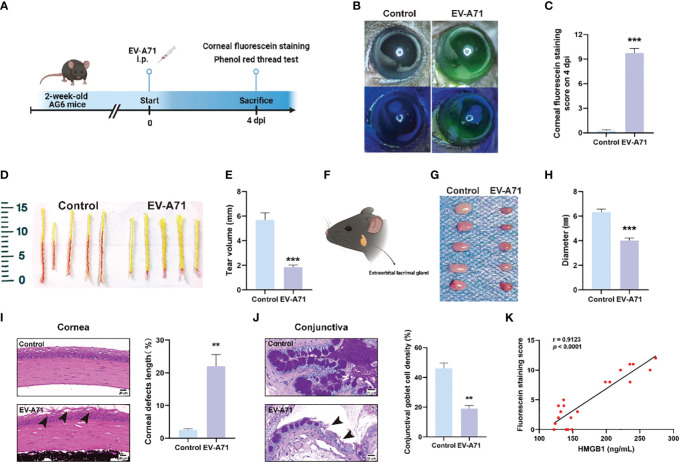
EV-A71 induced reduction of tear volume and the abnormality of LGs. **(A)** Two-week-old AG6 mice were inoculated with EV-A71 at 10^7^ PFU/mouse. **(B)** Representative image of fluorescein sodium staining. **(C)** Corneal fluorescein score after EV-A71 infection (n = 8). **(D, E)** Tear production at 4 dpi was measured by phenol red-impregnated cotton threads (n=10). **(F)** Location of the extraorbital lacrimal gland. **(G)** LGs from five mice were isolated on 4 dpi. **(H)** There was statistical significance in morphometric characteristics for the lacrimal gland diameters measured between the two groups (n=5). **(I)** Representative H&E staining images of corneal sections and quantitative analysis of the corneal epithelial defects (n = 3). Arrow: desquamated corneal epithelium. **(J)** Photographs of PAS staining of the conjunctiva showed the density of goblet cells and quantitative analysis of goblet cell density in the conjunctiva (n = 3). Arrow: loss of goblet cells. **(K)** The correlation between corneal fluorescence sodium dyeing and HMGB1 (n = 20). Data were presented as mean ± SEM of three independent experiments. (**p < 0.01, ***p < 0.001).

We established a mouse model of EV-A71 infection by intraperitoneal EV-A71 infection (**Figure 1A**), as previously described (You et al., 2023). The fluorescence staining of the corneal epithelium was observed before injection and at 4 dpi in mice (**Figures 1B, C**), indicating the tear film instability and the desiccation of the ocular surface. Phenol red cotton threads were also used to assess tear secretion and it in EV-A71-infected mice decreased greatly than the normal control levels (**Figures 1D, E**). Then, the extraorbital lacrimal glands (hereinafter called lacrimal glands) were isolated for further investigated (**Figure 1F**) and our results showed that the lacrimal glands of infectious mice were statistically smaller compared to the control ones (**Figures 1G, H**). Decreased tear secretion or an imbalance in tear film homeostasis would lead to Corresponding damages to corneal epithelium cells and Goblet cells of conjunctiva (Li et al., 2022). We then conducted histomorphology assessments to investigate the structural and morphological changes in corneas and conjunctival goblet cells after infection. The corneal structure and cell morphology were first evaluated by H&E staining. The normal corneal cells were densely and neatly arranged without inflammatory cells infiltration. However, the corneal superficial epithelial layers turned disrupted and desquamated in EV-A71-infected group. The rough area of cornea in EV-A71-infected group was markedly increased about 20% compared to the normal group, with a statistically significant difference (**Figure 1I**). Next, goblet cells were specialized cells that secrete mucins to lubricate the ocular surface, and its loss could aggravate dry eye (Alam et al., 2020). As shown in **Figure 1J**, the PAS‐positive goblet cells are abundantly presented in the conjunctival fornix of the normal cornea. However, goblet cells of EV-A71-infected group were lost distinctly, and the average density of stained cells in the conjunctiva significantly reduced 20% compared with the control group. Finally, we investigated the relationship between the systemic inflammation and corneal injury. The fluorescein staining indicated the extent of cornea damage (Fonn et al., 2010) and the serum level of HMGB1 was applied as an indicator for the severity of infection (Zheng et al., 2017). Pearson correlation analysis showed a positive correlation between corneal fluorescein scores and HMGB1 levels (**Figure 1K**). Thus, these results suggest that EV-A71 infection could lead to ocular surface desiccation and lacrimal gland changes. We hope to determine the causes of the symptoms by carrying out research on corneas and lacrimal glands in the following studies.

### 3.2 EV-A71 did not cause pathological changes in cornea

**Figure 2:**
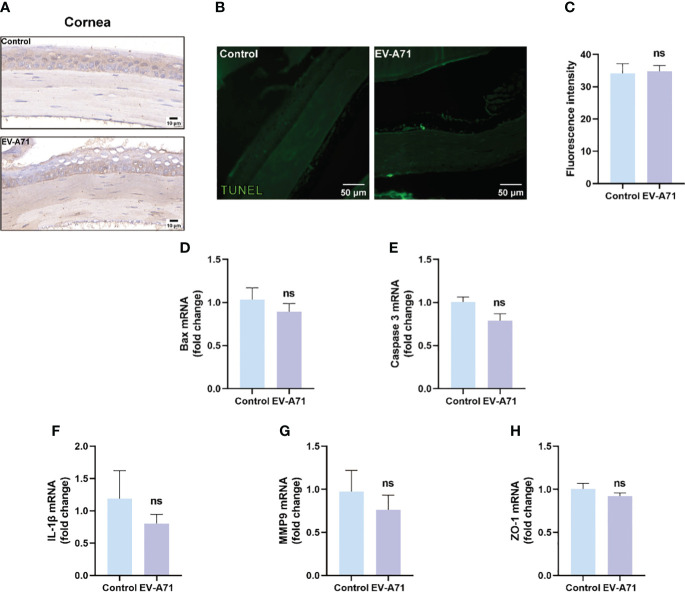
EV-A71 infection had no direct effect on corneas. **(A)** IHC with EV-A71 antibody was used for identification of the virus in corneas. **(B)** Representative images of TUNEL staining in corneas. **(C)** Quantification of fluorescence intensity (n = 3). **(D, E)** qRT-PCR were performed to detect bax and caspase 3 mRNA expression (n = 4). **(F–H)** The mRNA expression of IL-1β, MMP9, and ZO-1 was quantified using RT-PCR (n = 4). Data were presented as mean ± SEM of three independent experiments. (ns, not significant).

It is known that corneal epithelium lesions can lead to dry eye symptoms such as a decrease in tear film stability and tear secretion (Feng and Zhang, 2023), thus we investigated the condition of inflammation and apoptosis in the corneas. We first explored whether EV-A71 existed in corneas by IHC and there was no evidence of virus existence in corneas of EV-A71-infected mice (**Figure 2A**). Our findings suggest that no apoptosis was observed in the corneas of infectious group using TUNEL assay (**Figures 2B, C**). Apoptosis marker bax and caspase-3 were also evaluated by qRT-PCR which further validated the former result (**Figures 2D, E**). Then, we assessed the mRNA expression levels of inflammatory cytokines in cornea. The qRT-PCR results revealed that, compared to the control group, infectious mice had equal mRNA expression levels of IL-1β and MMP9 in the corneas (**Figures 2F, G**). ZO-1, as a marker of corneal epithelial tight junction, are expressed in the superficial cell layer of the corneal epithelium (Yang et al., 2018). The decreased expression of ZO-1 indicated the corneal epithelial barrier disruption in ocular surface disease. We investigated the expression of ZO-1 in corneal epithelium using qRT-PCR, and found that the expression of ZO-1 showed no clear difference between the two groups (**Figure 2H**). These results, taken together, suggested that cornea should not assume main responsibility for dry eye symptoms.

### 3.3 RNA seq analysis of murine corneas upon EV-A71 infection

**Figure 3:**
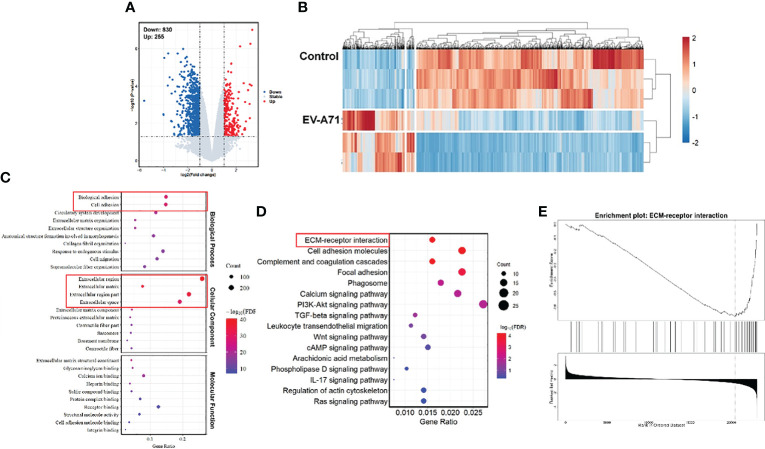
Comparison of gene expression profiles of the corneas from EV-A71 infected mice and controls. **(A)** The volcano plot of the DEGs in the corneas from control and EV-A71-infected mice (n = 3). **(B)** The heat map of the DEGs between these two groups. **(C)** The top 30 enriched functions from GO analysis of DEGs. Red rectangle indicating the significantly enriched GO terms. **(D)** DEG enriched pathways from KEGG analysis. **(E)** GSEA enrichment analysis of the differentially expressed genes. GSEA, Gene Set Enrichment Analysis.

We further carried out RNA-Seq analysis. In total, we obtained 36469 gene features from our sequencing study. There were 1085 differentially expressed genes (DEGs) in corneas from infectious mice compared to that from the control ones (fold change log2(fc) ≥ 0.5, p < 0.05). Of the 1085 DEGs, 255 were up-regulated and 830 were down-regulated. The volcano plot (**Figure 3A**) and heat map (**Figure 3B**) were displayed all the DEGs. As displayed in **Figure 3C**, GO analysis displayed that the differentially expressed genes were associated with extracellular region, extracellular matrix, extracellular space, extracellular region part, biological adhesion and cell adhesion. The top 16 significantly enriched KEGG pathways are described in **Figure 3D**. KEGG pathway analysis revealed top 4 enriched pathways among these DEGs, including ECM-receptor interaction, Cell adhesion molecules, Complement and coagulation cascades, and Focal adhesion. Although the virus infection did not cause significant pathological damage on corneas, severe tear deficiency caused changes in the corneal genomic level, especially the downregulation of the ECM-receptor interaction (**Figure 3E**).

### 3.4 EV-A71 infection induced functional and pathologic changes in lacrimal gland

**Figure 4:**
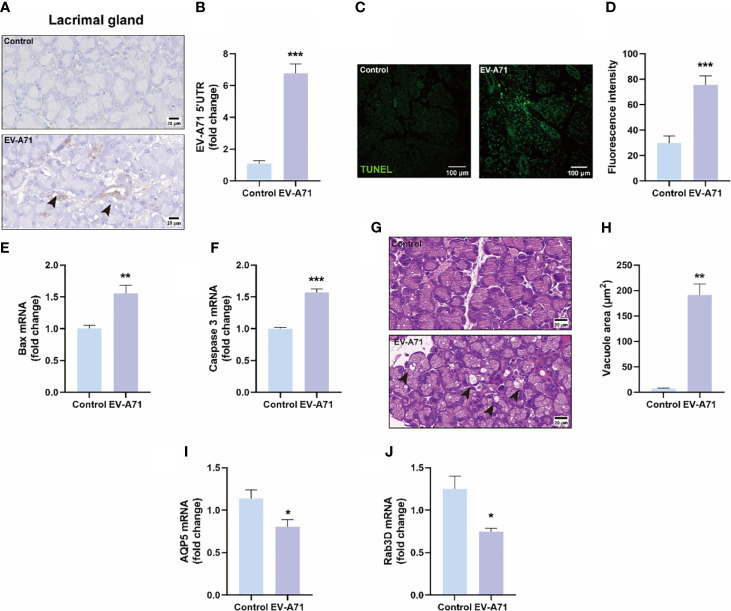
Evaluation of EV-A71 expression and apoptosis level in lacrimal gland with and without infection. **(A)** IHC was used to observe the virus expression. **(B)** EV-A71 expression was assessed post using qRT-PCR (n = 7). **(C)** The apoptotic injury in the lacrimal gland was evaluated using TUNEL. **(D)** Quantification of fluorescence intensity (n = 3). **(E, F)** qRT-PCR were performed to detect bax and caspase 3 mRNA expression (n = 4). **(G)** H&E staining of the lacrimal gland. Arrow: increased large vacuoles within acinar cells. **(H)** Quantitative analysis of vacuolar size within acinar cells (n = 3). **(I)** AQP5 signals in lacrimal glands detected by qRT-PCR (n = 4). **(J)** Rab3D expression in lacrimal glands detected by qRT-PCR analysis (n = 4). Data were presented as mean ± SEM of three independent experiments. (*p < 0.05, **p < 0.01, ***p < 0.001).

We further explored whether the appearance of ocular surface symptoms was related to lacrimal glands. The presence of EV-A71 infection in lacrimal glands were confirmed using IHC and qRT-PCR (**Figures 4A, B**). We also observed an increased number of apoptotic cells in lacrimal glands of infectious mice compared with control ones (**Figures 4C, D**). The expression of apoptosis-associated genes, such as bax and caspase-3, were upregulated in the EV-A71-infected group (**Figures 4E, F**). Then, we found that the acini in the healthy gland exhibited uniformity in size, neatness in arrangement, and normality in morphology while more large vacuoles were observed in infected group (arrows) (**Figures 4G, H**). This may be an indication that EV-A71 infection can cause the dysfunction of the excretion of tear fluid from the LGs, subsequently decreasing tear drops. To further investigate the alterations in the lacrimal gland, we evaluated the expression level of AQP5, a water channel protein, in the lacrimal gland (Cao et al., 2023). Our data showed that AQP5 expression in the lacrimal glands of EV-A71-infected mice was decreased compared with that in the controls (**Figure 4I**). Moreover, Rab3D, a specific marker of secretory vesicle maturation (Meng et al., 2016), was downregulated in the lacrimal glands with EV-A71 infection, as shown by qRT-PCR (**Figure 4J**).

### 3.5 RNA-seq analysis revealed the activation of pro-inflammatory signaling pathways in lacrimal glands from EV-A71-infected mice

**Figure 5:**
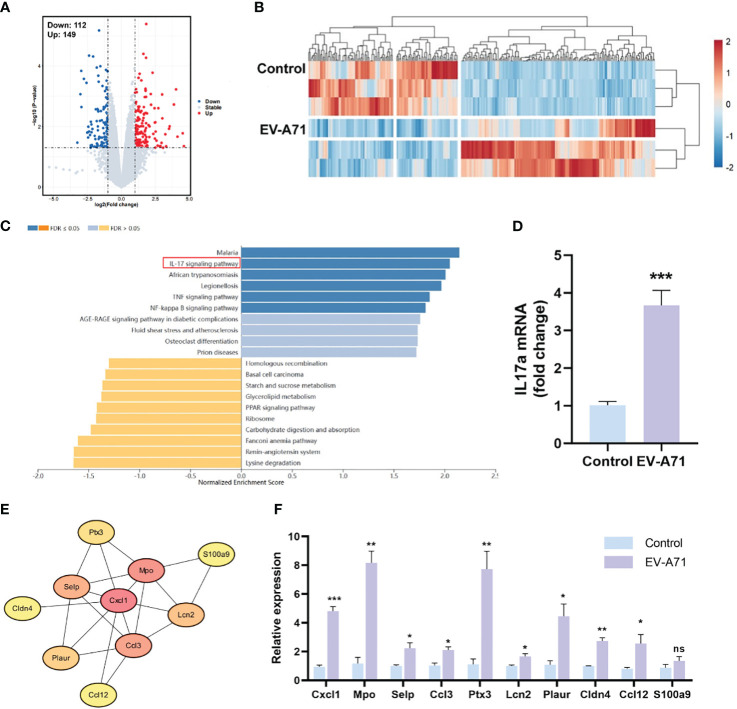
Expression profiles of the LGs in HFMD mice and controls. **(A, B)** The volcano plot and heatmap of DEGs. Red indicates high expression and blue represents low expression. **(C)** KEGG pathway analysis for the DEGs. **(E)** The PPI network image shows the top 10 highest degree genes calculated by Cytoscape. PPI, Protein–protein interaction. **(D, F)** mRNA expression level of IL-17a, Cxcl1, Mpo, Selp, Ccl3, Ptx3, Lcn2, Plaur, Cldn4, Ccl12, and S100a9. Data are presented as the mean ± SEM (n = 4). Data were presented as mean ± SEM of three independent experiments. (ns, not significant, *p < 0.05, **p < 0.01, ***p < 0.001).

We then compared the transcriptomic profiles of the LGs in infected and control mice. As shown in **Figures 5A, B**, the analysis identified 261 DEGs between the two groups, with 149 genes being upregulated and 112 showing downregulation. GO analysis revealed that the DEGs were enriched in the biological processes of response to external stimulus, defense response, response to external biotic stimulus, and the cellular component of extracellular region, extracellular space, extracellular region part (**Supplementary Figure 1**). In KEGG analysis, the results indicated that genes were mainly associated with IL-17 signaling pathway, TNF signaling pathway and NF-κB signaling pathway, which were significantly up-regulated (**Figure 5C**). The mRNA expression of IL-17a was assayed by using RT-qPCR and it was significantly increased in the EV-A71-infected group compared with that of control group (**Figure 5D**). STRING database analysis identified 63 nodes and 29 edges and Cytoscape analysis identified ten top hub genes, including Cxcl1, Mpo, Selp, Ccl3, Ptx3, Lcn2, Plaur, Cldn4, Ccl12, and S100a9 (**Figure 5E**). Using qRT-PCR to validate 10 core shared genes identified by GSEA analysis and the results showed that the expression of them could be up-regulated after EV-A71 infection (**Figure 5F**).

### 3.6 Tear film and ocular surface parameters were assessed with K5M and inflammatory factor was upregulated in HFMD patients

**Figure 6:**
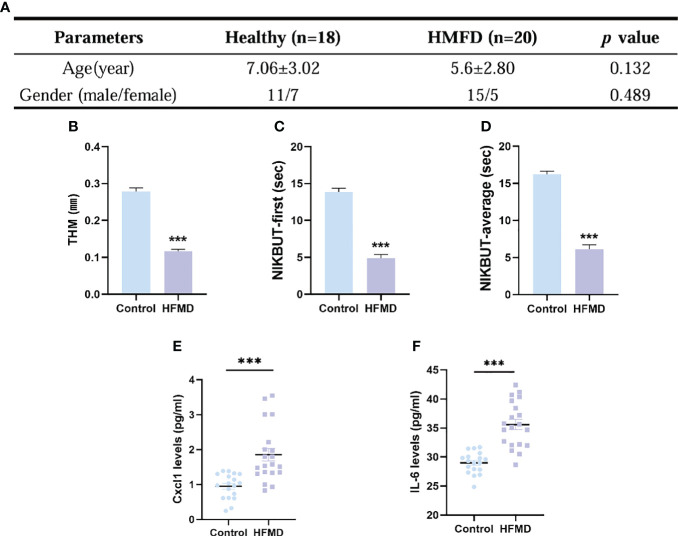
Evaluation of dry eye parameters and tear inflammatory factors levels in pediatric patients with HFMD (EV-A71). **(A)** No significant difference existed in the age and gender between the two groups. **(B–D)** TMH, tear meniscus height; NIKBUT, first and average non-invasive Keratograph breakup time. **(E, F)** Levels in tear soluble factors in study subjects between the two groups. Data were presented as mean ± SEM of three independent experiments. (***p < 0.001).

A total of 38 subjects (18 healthy and 20 HFMD) were identified. There was no significant difference between the two groups in age (p = 0.132) and gender (p = 0.489) (**Figure 6A**). The TMH value in eyes of the HFMD group was lower than that in the eyes of the control group (**Figure 6B**). The NIKBUT-f and NIKBUT-a of the HFMD was significantly shorter than the control group (**Figures 6C, D**). Tear fluid samples were collected to assess tear inflammatory factor levels from the study subjects. The levels of pro-inflammatory factors Cxcl1 and IL-6 were observed to be significantly increased in HFMD patients compared to healthy controls (**Figures 6E, F**). Although most of the children patients stated no obvious ocular discomfort, our examination found a significant reduction in tear stability and increased inflammation cytokines concentration in tears.

## 4 Discussion

**Figure 7:**
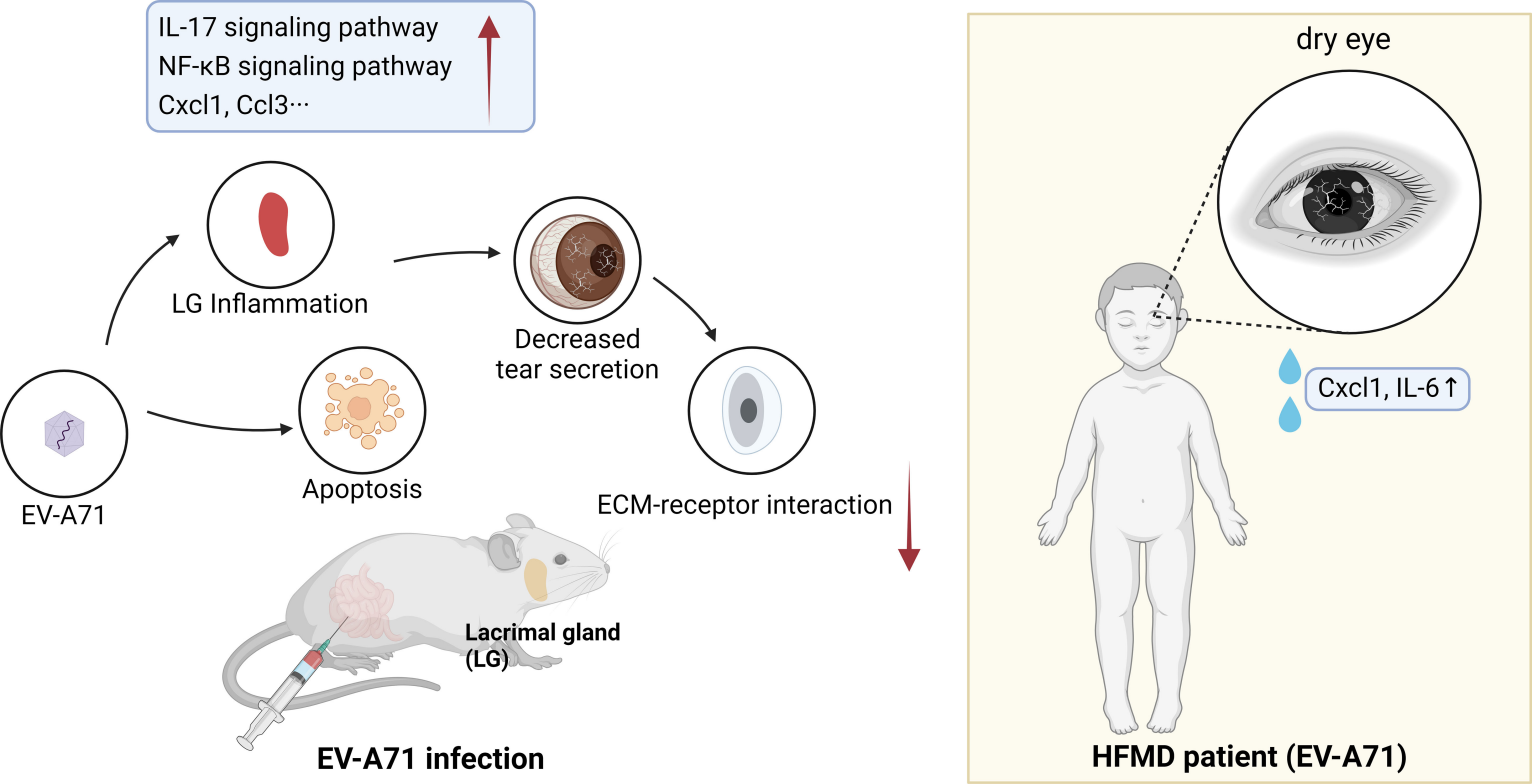
Schematic diagram of the possible mechanisms of HFMD-induced dry eye.

We found that EV-A71 infected mice developed dry eye-like symptoms such as reduced tear secretion. We have studied the cornea and lacrimal glands and found that the account for this symptom was that viral infection leads to apoptosis and increased inflammation in lacrimal glands, and the function of lacrimal glands were impaired. We then performed relevant tests on the patients to help verify the experimental results (**Figure 7**).

HFMD, a childhood viral disease initiated by enteroviruses (EVs), can lead to aseptic meningitis, acute flaccid paralysis, and even neurogenic pulmonary edema (Yang et al., 2022). However, there is little research regarding the effects of HFMD on eyes. This is the first study to explore the ocular surface damage and its underlying mechanisms in HFMD. Here, we show that when suffering from HFMD, pediatric patients exhibited symptoms of tear film instability and dry eye. Then we selected experimental mice model to explore the causes of dry eye symptoms. Finally, we identified that EV-A71 infection activated the intestinal and lacrimal gland inflammation, resulting in decreased aqueous tear secretion and dry eye-like changes in the ocular surface. Our study significantly enhances the understanding of hand, foot, and mouth disease (HFMD) ocular manifestations, a subject previously limited to isolated clinical reports on conditions like optic neuritis and maculopathy. Unlike these earlier studies, our research identifies a novel link between HFMD and dry eye symptoms due to lacrimal gland damage, marking an important addition to the known spectrum of HFMD-related ocular complications. Our methodological approach, blending clinical observations with an EV-A71-infected mouse model, sheds light on the inflammatory processes involved, offering a comprehensive perspective on HFMD’s systemic impacts and guiding future clinical practice toward early detection and management of ocular symptoms.

Our dual approach, employing clinical observations alongside a controlled animal model, has enabled a comprehensive exploration of this phenomenon. The robustness of our methodology lends substantial credibility to our findings, ensuring that the observed effects are not mere coincidences but are intricately connected to the pathophysiology of HFMD. The implications of this discovery extend far beyond the realms of academic interest. Clinically, it opens new avenues for the management of HFMD, especially in pediatric patients, where ocular health can now be monitored more vigilantly. The early detection of dry eye symptoms in HFMD could lead to more timely and targeted interventions, potentially improving patient outcomes. Furthermore, our study contributes significantly to the ongoing discussions about the systemic impacts of viral infections. By establishing a clear connection between a common viral illness and ocular surface complications, our research underscores the need for a holistic approach in managing infectious diseases. In conclusion, this research not only fills a critical gap in our understanding of HFMD but also sets a precedent for future studies exploring the ocular manifestations of systemic diseases. The insights gained from this study could pave the way for innovative treatment strategies, enhancing the quality of life for those affected by this widespread disease.

Despite its groundbreaking findings, our study is constrained by the small patient sample size and lack of longitudinal monitoring of ocular surface changes throughout HFMD progression. To further this field, future research should focus on dynamic monitoring in group settings like schools, comprehensive prognostic studies for long-term outcomes, development of therapeutic interventions targeting lacrimal gland protection, and expansion of patient demographics to understand HFMD’s ocular effects across diverse populations. These steps are crucial for translating our findings into effective clinical applications and enhancing the quality of life for those impacted by HFMD.

## Data availability statement

The RNA-seq data presented in the study are deposited in the Sequence Read Archive (SRA) repository, accession number PRJNA1071792. The original contributions presented in the study are included in the article/**Supplementary Material**, further inquiries can be directed to the corresponding author.

## Ethics statement

This study was conducted in the Department of Ophthalmology, The Affiliated Drum Tower Hospital, Medical School of Nanjing University. This study was approved by the Ethics Committee of Children’s Hospital of Nanjing Medical University (Application No.202311015-1). The studies were conducted in accordance with the local legislation and institutional requirements. Written informed consent for participation in this study was provided by the participants’ legal guardians/next of kin. All experimental procedures in animals were approved by the Animal Care and Use Committee of Nanjing University and were carried out in accordance with the Association for Research in Vision and Ophthalmology (ARVO) Statement for the Use of Animals in Ophthalmic and Vision Research. The study was conducted in accordance with the local legislation and institutional requirements.

## Author contributions

NZ: Writing – original draft, Data curation, Conceptualization. TC: Writing – original draft, Methodology, Formal analysis. QY: Writing – original draft, Validation, Software. DC: Writing – original draft, Visualization, Validation. LL: Writing – review & editing, Resources, Investigation. KH: Writing – review & editing, Supervision, Project administration, Investigation, Funding acquisition.
