# Supplementary figures and images for: Enterovirus A71 infection-induced dry eye-like symptoms by damaging the lacrimal glands
Source: Front Cell Infect Microbiol. 2024 Apr 2;14:1340075. doi: 10.3389/fcimb.2024.1340075 (PMC11018897; doi:10.3389/fcimb.2024.1340075)

**Supplementary Figure 1. GO analysis of cornea.**


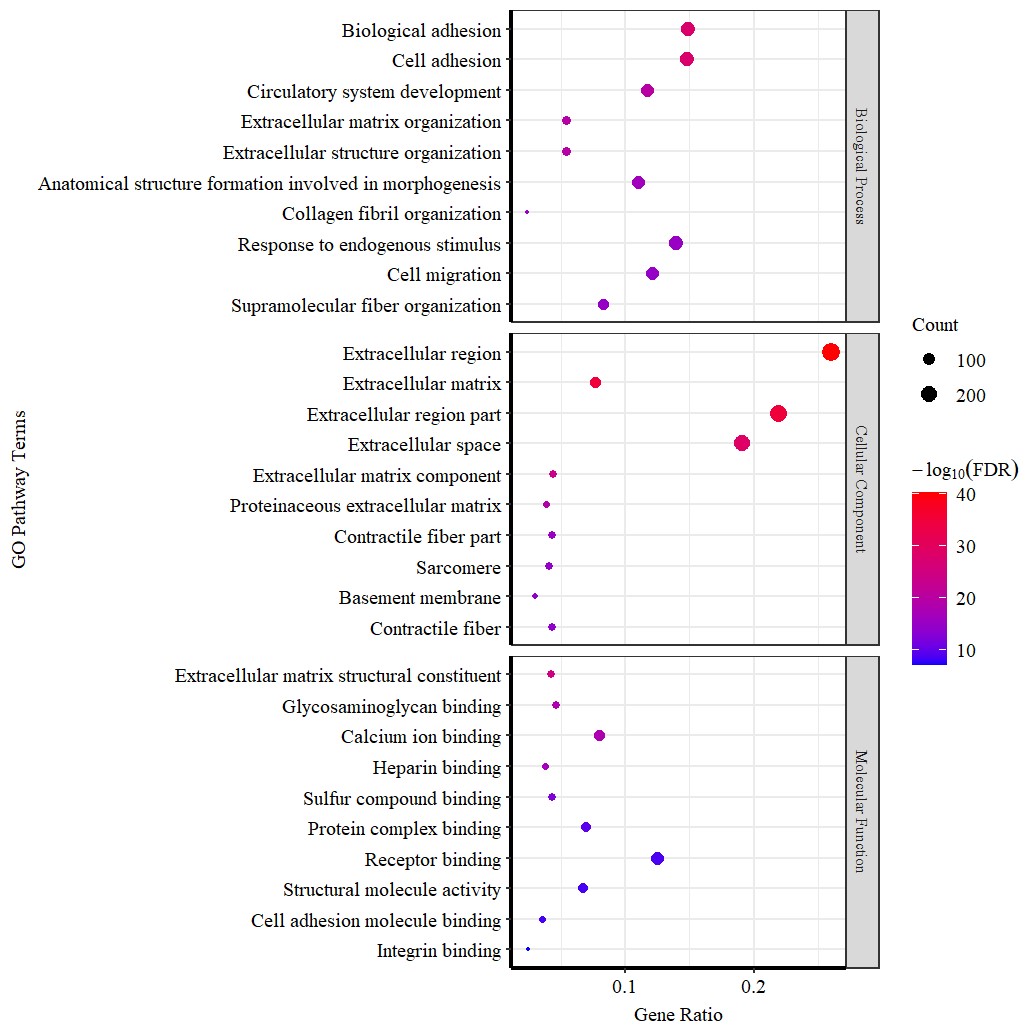

Supplement: Supplementary file 1 [file DataSheet_1.docx]
