# Supplementary material for: Enterovirus A71 infection-induced dry eye-like symptoms by damaging the lacrimal glands
Source: Front Cell Infect Microbiol. 2024 Apr 2;14:1340075. doi: 10.3389/fcimb.2024.1340075 (PMC11018897; doi:10.3389/fcimb.2024.1340075)
Supplement: Supplementary file 2 [file Table_1.docx]

**Supplementary Table 1. Primer Sequences**

| **Gene Name** | **Forward Primer** | **Reverse Primer** |
| --- | --- | --- |
| **EV71 5’UTR** | TAACTGCGGAGCACATACCC | ACGGACACCCAAAGTAGTCG |
| **Mouse GAPDH** | TGATGACATCAAGAAGGTGGTGAAG | TCCTTGGAGGCCATGTGGGCCAT |
| **ZO-1** | ACCACCAACCCGAGAAGAC | CAGGAGTCATGGACGCACA |
| **IL-17a** | CGCAATGAAGACCCTGATAGAT | CTCTTGCTGGATGAGAACAGAA |
| **AQP-5** | ATCTACTTCACCGGCTGTTCC | GTCAGCTCGATGGTCTTCTTC |
| **Bax** | TGAAGACAGGGGCCTTTTTG | AATTCGCCGGAGACACTCG |
| **CASP-3** | ATGGAGAACAACAAAACCTCAGT | TTGCTCCCATGTATGGTCTTTAC |
| **Rab3D** | CGAGATCCACGTGTCGGAAG | CACTAGCGGATGCCATCTCA |
| **Cxcl1** | GCCTATCGCCAATGAGCTG | TGGGGACACCTTTTAGCATC |
| **Mpo** | AGTTGTGCTGAGCTGTATGGA | CGGCTGCTTGAAGTAAAACAGG |
| **Selp** | CATCTGGTTCAGTGCTTTGATCT | ACCCGTGAGTTATTCCATGAGT |
| **Ccl3** | TTCTCTGTACCATGACACTCTGC | CGTGGAATCTTCCGGCTGTAG |
| **Ptx3** | CCTGCGATCCTGCTTTGTG | GGTGGGATGAAGTCCATTGTC |
| **Lcn2** | TGGCCCTGAGTGTCATGTG | CTCTTGTAGCTCATAGATGGTGC |
| **Plaur** | TGTGAGAGTAACCAGAGCTGC | CCGAAGCACGGTAGTCCTG |
| **Cldn4** | GTCCTGGGAATCTCCTTGGC | TCTGTGCCGTGACGATGTTG |
| **Ccl12** | ATTTCCACACTTCTATGCCTCCT | ATCCAGTATGGTCCTGAAGATCA |
| **S100a9** | ATACTCTAGGAAGGAAGGACACC | TCCATGATGTCATTTATGAGGGC |
